# Temporal characteristics of NF-κB inhibition in blocking bile-induced oncogenic molecular events in hypopharyngeal cells

**DOI:** 10.18632/oncotarget.26917

**Published:** 2019-05-21

**Authors:** Panagiotis G. Doukas, Dimitra P. Vageli, Sotirios G. Doukas, Clarence T. Sasaki

**Affiliations:** ^1^ The Yale Larynx Laboratory, Department of Surgery, Yale School of Medicine, New Haven, CT, USA

**Keywords:** NF-κB inhibition, STAT3, miR-21, bile reflux, hypopharyngeal cancer

## Abstract

Biliary esophageal reflux at acidic pH is considered a risk factor in laryngopharyngeal cancer. We previously showed the key role NF-*κ*B in mediating acidic bile-induced pre-neoplastic events in hypopharyngeal cells, and that co-administration of specific NF-*κ*B inhibitor, BAY 11-7082, together with acidic bile, can effectively prevent its related oncogenic molecular effects. We hypothesize that the addition of BAY 11-7082 (10μM) either before or after application of acidic bile (400μM conjugated bile acids; pH 4.0), is capable of comparably blocking acidic bile-induced oncogenic molecular phenotypes in murine hypopharyngeal primary cells. We performed immunofluorescence, luciferase assay, western blot and qPCR analysis, demonstrating that 15-min of pre- or post-application of BAY 11-7082 effectively inhibits acidic bile-induced NF-*κ*B activation, transcriptional activation of *RELA(p65), STAT3, EGFR, IL-6, bcl-2,* W*NT5A*, “upregulation” of “oncomirs” miR-21, miR-155, miR-192 and “downregulation” of “tumor suppressor” miR-34a, miR-375, miR-451a. Our observations support the understanding that acidic bile-induced deregulation of anti-apoptotic or oncogenic factors, *bcl-2*, *STAT3, EGFR, IL-6, WNT5A*, miR-21, miR-155, miR-375, is highly NF-*κ*B-dependent, showing that even post-application of inhibitor can suppress their deregulation. In conclusion, application of specific NF-κB inhibitor, has the capability of adequately blocking the early oncogenic molecular events produced by acidic bile whether it is applied pre or post exposure. In addition to therapeutic implications these findings provide a window of observation into the complex kinetics characterizing the mechanistic link between acidic bile and early neoplasia. Although BAY 11-7082 itself may not be suitable for clinical use, the application of other NF-κB inhibitors merits exploration.

## INTRODUCTION

Laryngopharyngeal reflux (LPR) has been linked to chronic inflammatory and neoplastic diseases of the upper aero-digestive tract [1]. Although several known risk factors have been associated with laryngopharyngeal cancer, the presence of duodenogastric or bile fluid in patients with LPR suggests the possible carcinogenic effect of mixed (acid and bile) refluxate on hypopharyngeal mucosa [2–4]. In this connection, we have previously shown that acidic bile is capable of upregulating NF-κB signaling and transcriptionally activating oncogenic factors while deregulating cancer-related miRNA markers in exposed hypopharyngeal primary cells [5–7]. Furthermore, we showed that these alterations are early molecular events that are specifically linked to premalignant histopathological changes, such as abnormal hyperplasia and dysplasia, seen in exposed murine hypopharyngeal mucosa [8, 9]. Furthermore, our recent findings document that co-administration of NF-κB inhibitor BAY 11-7082 with acidic bile is capable of inhibiting the upregulation of NF-κB signaling and deregulation of oncogenic mRNA and miRNA phenotypes [6, 7, 10].

We hypothesize that treatment of hypopharyngeal cells with BAY 11-7082 before or after acidic bile exposure (pre- and post-treatment) may have effects comparable to its co-administration with acidic bile in inhibiting its oncogenic mRNA and miRNA phenotypes [6, 7]. In using murine hypoharyngeal primary cells we performed a series of assays according to a previously established *in vitro* model to highlight the effects of pre- or post-application of BAY 11-7082 on acidic bile-induced cancer-related molecular alterations [6, 7]. Although BAY 11-7082 itself may not be suitable for clinical use, this pre-clinical *in vitro* exploration is intended to conceptually support and encourage the future clinical use of NF-κB inhibition in preventing the tumorigenic effects of biliary reflux disease.

## RESULTS

### Pre-or post-application of BAY 11-7082 comparably prevents acidic bile-induced NF-κB nuclear translocation and bcl-2 overexpression

**Figure 1:**
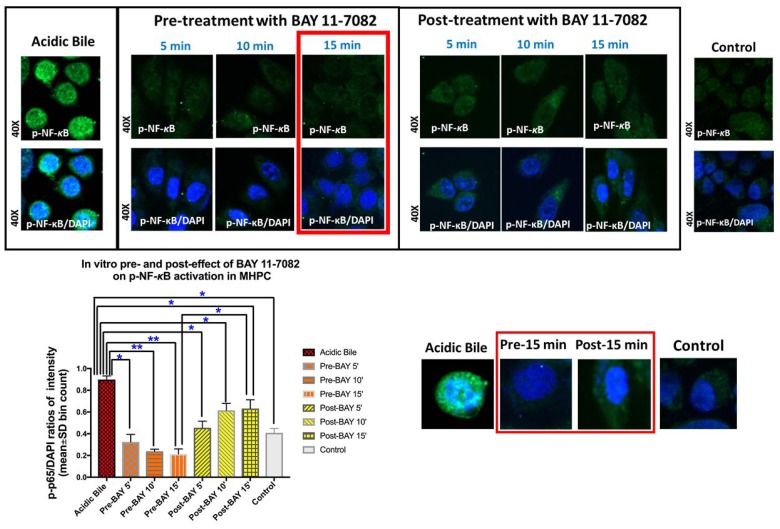
Pre- or post-application of BAY 11-7082 inhibits the acidic bile-induced nuclear translocation of phospho-NF-*κ*B in MHPC. Immunofluorescence staining for phospho-NF-*κ*B (p-p65 S536) reveals that 5, 10, or 15 min either of pre- or post-application of BAY 11-7082 inhibits acidic bile-induced p-NF-*κ*B nuclear translocation, demonstrating decreased p-NF-κB nuclear staining and significantly reduced p-NF-κB nuclear expression (p-p65/DAPI ratios of intensity; mean±SD; bin count), compared to MHPC exposed to acidic bile alone. Pre-application of NF-κB inhibitor induces a more intense effect than post-application. Fifteen minutes of pre-application is found to be significantly more effective than its post-application, demonstrating significantly lower nuclear p-NF-κB (p-p65/DAPI) intensity, while no significant differences of the nuclear p-NF-κB (p-p65/DAPI) intensity are observed between 5 or 10 min of pre- and post-application. Control treated-group present a weak p-NF-κB staining [green: p-p65 (S536); blue: DAPI for nuclear staining; *p* values by *t* test; multiple comparisons by Holm-Sidak; GraphPad Prism 7.0; p-p65/DAPI ratios of intensity evaluated by Zen imaging software; Zeiss Microscopy].

We observed that either pre- or post-applications of NF-κB inhibitor, successfully inhibited acidic bile-induced NF-κB activation in treated MHPC. However pre-application was found more effective in reducing both the nuclear translocation and cytoplasmic accumulation of p-NF-κB. Specifically, immunofluorescence assay (IF) revealed that MHPC treated with NF-κB inhibitor 15 min before acidic bile application (pH 4.0) demonstrated decreased p-p65 nuclear staining, implying that BAY 11-7082 blocked acidic bile-induced p-p65 translocation to the nucleus (Figure 1). (*p* < 0.05; by paired *t* test; GraphPad Prism 7.0). Fifteen minutes of post-application of BAY 11-7082 was also effective but was found less effective than its pre-application. No significant differences were observed between 5 or 10 min of pre- and post-application. Therefore, our protein, mRNA and miRNA analyses were focused on the 15 min pre- and post-treated groups, since 15 min seemed to be the minimum interval for visually detectable differences between the two groups.

**Figure 2:**
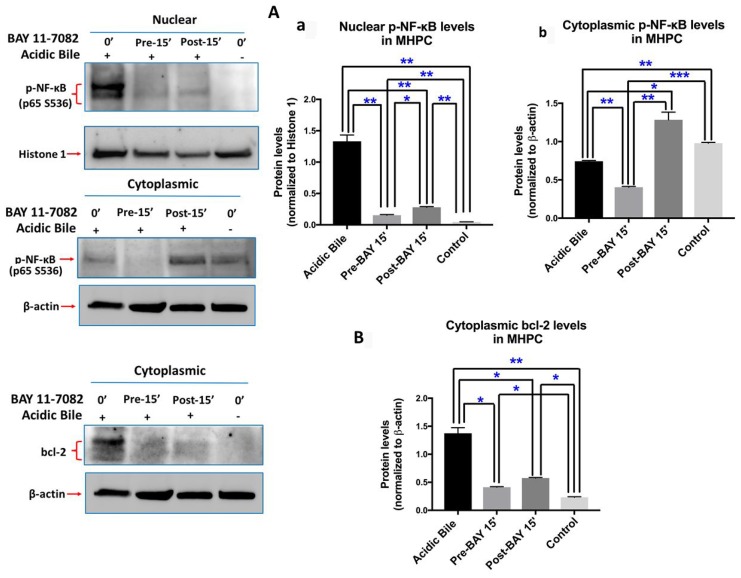
Pre- or post-application of ΒΑΥ 11-7082 inhibits acidic bile-induced NF-*κ*B activation and bcl-2 overexpression in MHPC. Western blot analysis is performed in nuclear and cytoplasmic protein extracts of MHPC for p-NF-*κ*B (p65 S529), and cytoplasmic bcl-2. MHPC exposed to acidic bile demonstrate a significant overexpression of nuclear p-p65 and cytoplasmic bcl-2, compared to controls. Pre- or post-application of BAY 11-7082 results in significantly (**A-a**) reduced nuclear p-p65 levels compared to acidic bile alone. Pre-application also results in significantly reduced (**A-b**) cytoplasmic p-p65 levels compared to acidic bile alone. However, post-application of BAY 11-7082 results in elevated cytoplasmic p-p65 levels compared to acidic bile alone. (**B**) Pre- or post-application of BAY 11-7082 results in significant reduction of cytoplasmic bcl-2 levels compared to acidic bile alone (Paired *t*-test, ^*^p < 0.05; ^**^p < 0.005; ^***^p < 0.0005; GraphPad Prism 7.0). (Mean ± SD of three independent experiments). (β-actin and Histone 1 are used for the normalization of cytoplasmic and nuclear protein extracts, respectively).

Western blot analysis confirmed this observation, demonstrating that pre-application of BAY 11-7082 resulted in reduced nuclear and cytoplasmic p-p65 levels, compared to cells treated with acidic bile alone (Figure 2A-a,b). Post-application of BAY 11-7082 was also found effective in inhibiting acidic bile-induced p-p65 nuclear translocation. This observation was characterized by significantly reduced nuclear p-p65 levels in MHPC post-treated with BAY 11-7082 compared to acidic bile alone. However, post-application of BAY 11-7082 resulted in an accumulation of cytoplasmic p-p65 (Figure 2A-b).

We also found by Western blot analysis that pre- or post-application with BAY 11-7082 suppressed acidic bile-induced cytoplasmic bcl-2 accumulation. This observation was characterized by a significant reduction of cytoplasmic bcl-2 levels in MHPC pre- or post-treated with BAY 11-7082 compared to acidic bile alone (Figure 2B). Pre-application was found slightly more effective in inhibiting acidic bile-induced bcl-2 overexpression compared to post-application.

Taken together, either pre- or post-application of BAY 11-7082 effectively prevented the acidic bile-induced NF-κB activation and bcl-2 overexpression, similar to that shown by the simultaneous application of BAY 11-7082 and acidic bile [6]. Pre-application with NF-κB inhibitor resulted in more intense inhibition of acidic bile-induced changes than post-application.

### Pre- or post-application of BAY 11-7082 effectively reduced acidic bile-induced NF-κB transcriptional activity in murine hypopharyngeal primary cells

**Figure 3:**
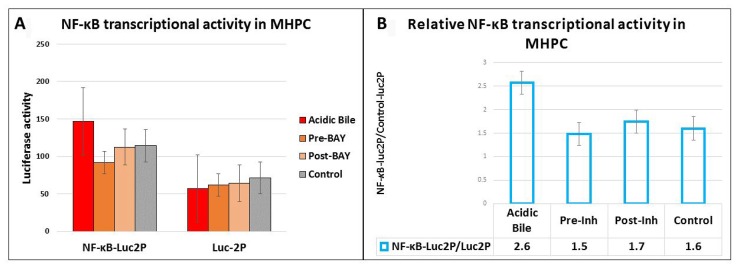
Luciferase assay demonstrates that either pre- or post-application of BAY 11-7082 prevents the acidic bile-induced NF-*κ*B transcriptional activity in MHPC. (**A**) Columns represent luciferase activity (mean ± standard error of two independent experiments) in MHPC transfected with control luciferase reporter (luc2P) and NF-*κ*B luciferase responsive element (NF-*κ*B-Luc2P). (**B**) Columns represent NF-*κ*B relative transcriptional activity in MHPC (NF-*κ*B-Luc2P/Luc2P: NF-*κ*B luciferase responsive element/ control luciferase reporter). Pre-Inh: 15 min of pre-application of BAY 11-7-082; Post-Inh: 15 min of post-application of BAY 11-7082.

We used an NF-κB luciferase assay to investigate the effect of pre- and post-application of BAY 11-7082 in preventing the NF-κB acidic bile-induced transcriptional activity in treated MHPC (Figure 3A). MHPC exposed to acidic bile alone induced higher levels of NF-κB transcriptional activity compared to neutral control. Pre- or post-treated MHPC with NF-κB inhibitor also resulted in a reduced transcriptional activity of NF-κB, compared to those treated with acidic bile alone (Figure 3B).

### Pre- or post-application of BAY 11-7082 Prevents the Acidic Bile-Induced mRNA Phenotype

**Figure 4:**
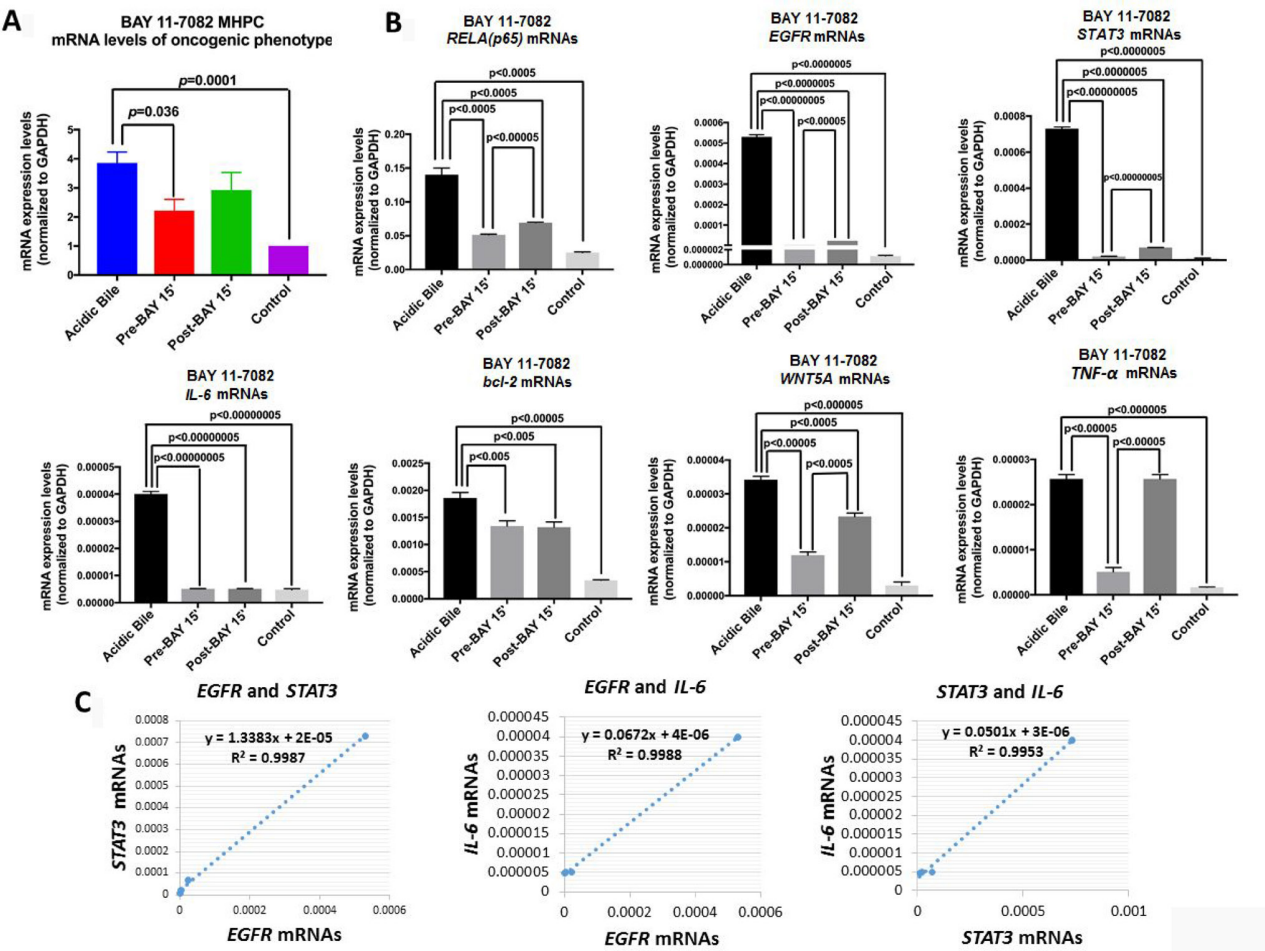
Pre- or post-application of BAY 11-7082 blocks the acidic bile-induced transcriptional activation of genes with oncogenic function in MHPC. (**A**) Transcriptional levels of the analyzed NF-***κ***B related genes with oncogenic function, are depicted in MHPC exposed to acidic bile alone and to (10 μM) BAY 11-7082 15 min before (Pre-BAY 15’) or after (Post-BAY 15’) acidic bile exposure, and controls. Graphs, created by GraphPad Prism 7 software (transcriptional levels of the analyzed genes are normalized to GAPDH by real time qPCR analysis). (ONE-WAY ANOVA, Freidman test). (**B**) Graphs represent transcriptional levels of each analyzed gene, *RELA*(*p65*)*, TNF-α, STAT3, IL-6, bcl-2, WNT5A,* and *EGFR* (relative to GAPDH reference gene), in experimental and control-treated MHPC. The data are derived from real-time qPCR analysis. (Data are derived from three independent experiments. Graphs, created by GraphPad Prism 7 software; by *t* test; multiple comparisons by Holm-Sidak). (**C**) Diagrams show significant linear correlations by Pearson analysis, between *EGFR* and *STAT3, EGFR* and *IL-6*, and *STAT3* and *IL-6* mRNAs (by Pearson analysis, *p* value < 0.05).

We performed qPCR to analyze the effect of pre- and post-application of BAY 11-7082 in preventing overexpression of NF-*κ*B and related oncogenic genes in acidic bile-treated MHPC (Figure 4). *RELA(p65), TNF-α, STAT3, EGFR, bcl-2*, *IL-6* and *WNT5A* were selected because they had been previously found to be overexpressed in the acidic bile-treated MHPC, and because their transcriptional activation was effectively prevented by simultaneous application of acidic bile with BAY 11-7082 [10].

We found that pre-application of NF-*κ*B inhibitor appeared to induce a more profound effect on the acidic bile-induced mRNA oncogenic phenotype, preventing its transcriptional activation (Figure 4A). This observation was characterized by significantly lower mRNA levels of *RELA(p65), TNF-α, STAT3, EGFR, bcl-2*, *IL-6* and *WNT5A* in MHPC pre-treated with NF-κB inhibitor compared to those exposed to acidic bile alone (Figure 4B). Although post-application of NF-*κ*B inhibitor induced a similar effect, its application resulted in a less significant reduction of the *RELA(p65), STAT3, and WNT5A* compared to its pre-application (Figure 4B). We also observed that transcriptional levels of *TNF-α* were only affected by pre-application of BAY 11-7082, however, anti-apoptotic *bcl-2* and cancer-related cytokine *IL-6* were similarly affected by pre- and post-application of BAY 11-7082, inducing significantly lower mRNA levels compared to acidic bile alone (*p* < 0.05; *t*-test, means ± SD; multiple comparisons by Holm-Sidak) (Figure 4B).

We used a Pearson analysis to identify correlations between NF-*κ*B inhibition-induced transcriptional levels of the analyzed genes. We found a significant linear correlation between mRNA ratios of *EGFR* and *STAT3* (*r =* 0.994385732, *p =* 0.0057)*, EGFR* and *IL-6* (*r =* 0.999416367, *p =* 0.0006), as well as *STAT3* and *IL-6* (*r =* 0.997638*, p =* 0.0024) in MHPC-treated groups (Figure 4C).

All the above support the observation that either pre- or post-application of NF-*κ*B inhibitor significantly prevented the acidic bile-induced transcriptional activation of NF-*κ*B and key oncogenic factors, as previously shown by the simultaneous application of BAY 11-7082 with acidic bile [6, 10]. However, pre-application resulted in a more profound inhibition of acidic bile-induced transcriptional changes compared to its post-application.

### Pre- or post-application of BAY 11-7082 Prevents the Acidic Bile-Induced miRNA Phenotype

**Figure 5:**
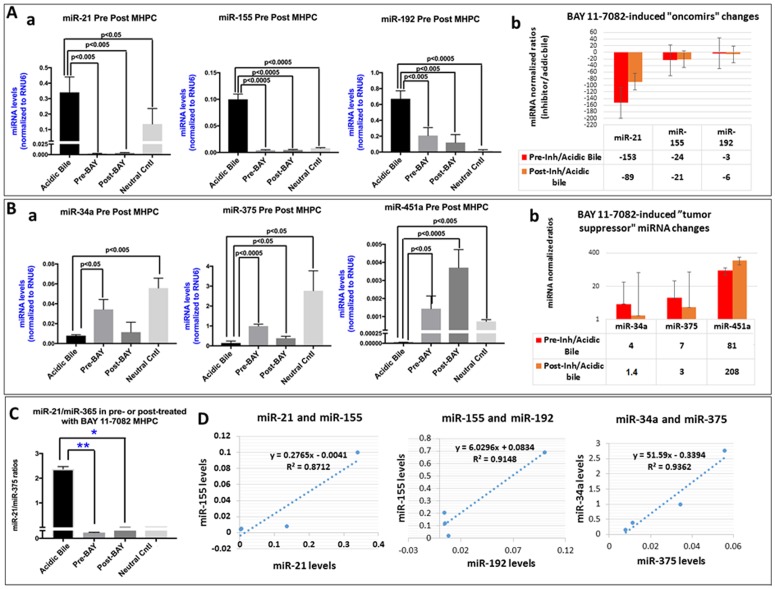
Pre- or post-application of BAY 11-7082 inhibits the acidic bile-induced deregulation of cancer-related miRNA markers, in MHPC. (**A**) Pre- or post-application of MHPC with BAY 11-7082 inhibits the acidic bile-induced (**a**) upregulation of the analyzed “oncomirs” miR-21, miR-155 and miR-192, demonstrated by significantly lower miRNA levels, compared to acidic bile-treated alone (*p* values by *t*-test; mean ±SD; multiple comparisons by Holm-Sidak; GraphPad Prism 7.0). (**b**) Graphs depict the BAY 11-7082-induced expression changes of each “oncomir” miR-21, miR-155 and miR-192 in MHPC (pre- or post-application relative to acidic bile alone) (normalization control: small RNA RNU6B). (**B**) Pre- or post-application of MHPC with BAY 11-7082 suppresses the acidic bile-induced (**a**) downregulation of the analyzed “tumor suppressor” miRNAs, miR-34a, miR-375, miR-451a, demonstrated by significantly higher acidic bile-alone (*p* values by *t*-test; mean±SD; multiple comparisons by Holm-Sidak; GraphPad Prism 7.0). (**b**) Graphs depict the BAY 11-7082-induced expression changes of each “tumor suppressor” miR-34a, miR-375 and miR-451a, in MHPC (Pre- or post-application relative to acidic bile alone) (Normalization control: small RNA RNU6B). (**C**) Pre- or post-application of BAY 11-7082 induces significantly lower miR-21/375 ratios in MHPC, compared to acdic bile alone (*p* values by *t*-test; mean ±SD; multiple comparisons by Holm-Sidak; GraphPad Prism 7.0)**.** (**D**) Diagrams show significant linear correlations by Pearson analysis, between miR-21 and miR-155, miR-155 and miR-192, and miR-34a and miR-375 (by Pearson analysis, *p* value < 0.05).

We performed a miRNA analysis to characterize the effect of pre- and post-application of BAY 11-7082 in preventing the alterations, of specific miRNA markers, under the stimulation by acidic bile (Figure 5). We analyzed the expression of “oncomirs” miR-21, miR-192, and miR-155 and “tumor suppressors” miR-451a, miR-34a, and miR-375, that were previously found to be deregulated in acidic bile-exposed premalignant murine laryngopharyngeal mucosa [9]. We found that either pre- or post-application of BAY 11-7082 comparably inhibited the acidic bile-induced upregulation of “oncomirs” (Figure 5A) and downregulation of “tumor suppressor” miRNAs (Figure 5B) in treated MHPC.

Specifically, we observed significantly lower levels of the analyzed “oncomirs” miR-21, miR-155 and miR-192 in pre- or post-treated with NF-*κ*B inhibitor cells compared to those exposed to acidic bile alone (*p*<0.05, *t* test; means±SD; multiple comparisons by Holm-Sidak) (Figure 5A-a). Pre-application of BAY 11-7082 was found to be more effective than post-application in preventing miR-21 overexpression, by inducing lower relative expression ratios (inhibitor/acidic bile) (Figure 5A-b)

In addition, either pre- or post-application of NF-κB inhibitor successfully inhibited the acidic bile-induced downregulation of “tumor suppressor” miR-34a and miR-375 and miR-451a (Figure 5B-a). However, pre-application of BAY 11-7082 was found to be more effective than post-application in preventing miR-34a and miR-375 downregulation, by inducing higher relative expression ratios (inhibitor/acidic bile) (Figure 5B-b), whereas post-application was found to result in a more intense inhibition of acidic bile-induced downregulation of “tumor suppressor” miR-451a in treated MHPC than post-treatment (Figure 5B-b). Finally, we found that the calculated miR-21/miR-375 ratio was significantly lower in either pre- or post-treated MHPC compared to acidic bile alone (*p*<0.05; ANOVA) (Figure 5C).

Pearson analysis revealed a significant positive correlation between BAY 11-7082-induced expression changes of “oncomirs” miR-21 and miR-155 (*r =* 0.933375, *p =* 0.0204), miR-155 and miR-192 (*r =* 0.956463, *p =* 0.0462), as well as of “tumor suppressor” miR-34a and miR-375 (*r =* 0.967551, *p =* 0.0325) (Figure 5D). We also observed a significant linear correlation between BAY 11-7082-induced expression changes of mRNA levels of NF-*κ*B transcription factor *RELA(p65)* and “oncomirs” miR-155 (*r =* 0.9181627, *p =* 0.0278) and miR-192 (*r =* 0.95775676, *p =* 0.0104) and a reverse correlation between mRNAs of *RELA(p65)* and ‘tumor suppressor” miR-34a (*r =* -0.8369361) in treated MHPC.

Taken together, miRNA analysis revealed that either pre- or post-application of NF-*κ*B inhibitor comparably and significantly prevented the acidic bile-induced deregulations of cancer-related miRNAs, as previously shown by the simultaneous application of BAY 11-7082 with acidic bile [7, 10].

## DISCUSSION

In patients with LPR, it is known that multiple reflux episodes may occur throughout the day. Since variable risk factors promote reflux events, the frequency and duration of events vary significantly within and across patients [11]. Because reflux events seem to be random in distribution, a treatment that requires a precise-temporal application synchronized to each reflux episode would be clinically impractical unless it could be demonstrated that effects of pre- or post-application of a treatment were largely equivalent and therefore could plausibly support a clinical regimen of topical pharmacologic management.

**Figure 6:**
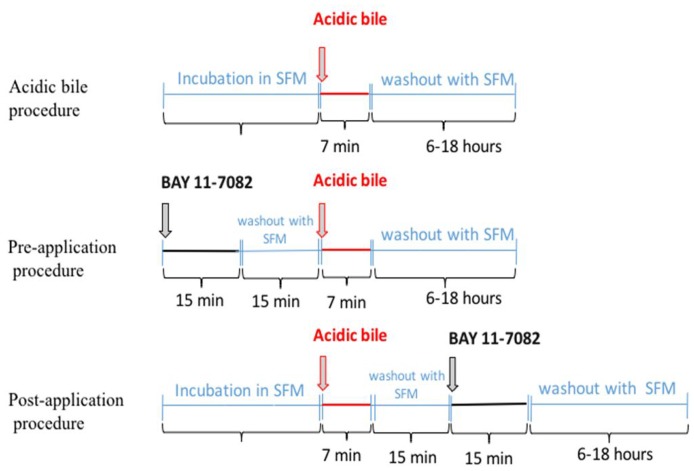
Schematic representation of pre- and post-application of NF-κB inhibitor (BAY 11-7082) in acidic bile-exposed MHPC.

In previous publications we documented the effectiveness of BAY 11-7082 as a strong NF-*κ*B inhibitor of acidic bile-induced early oncogenic mRNA and miRNA phenotypes in treated human and murine hypopharyngeal primary cells when applied concurrently with acidic bile [6, 7, 10]. Our current novel findings provide clear evidence that the application of BAY 11-7082 either before or after acidic bile exposure can also successfully inhibit the acidic-bile induced activation of NF-*κ*B and its related oncogenic mRNA and miRNA phenotypes. Our findings lead to the conclusion that targeted NF-*κ*B inhibition can either prevent or suppress the acidic bile oncogenic effect, whether it is administered before or after exposure to acidic bile. Although, NF-*κ*B inhibitor is applied for a short duration (15 min) within a 15 min time window before or after the 7-min application of acidic bile (Figure 6), it appears that both application models adequately suppress the acidic bile-induced oncogenic effect, demonstrating effectiveness of intermittent short duration therapy.

Οur findings also reveal that pre-application of NF-*κ*B inhibitor is significantly more effective than post-application in preventing the transcriptional activation of *RELA(p65)*, *STAT-3, EGFR, TNF-α* and *WNT5A*, further supporting the view that acidic bile-induced NF-*κ*B activation directly promotes the transcriptional activation of these genes, in agreement with our prior *in vitro* and *vivo* results [5, 6, 8, 10, 12]. It has been previously shown that BAY 11-7082 inhibits NF-κB, by inhibiting IκB-α phosphorylation and blocking proteosomal degradation of IκB-α, allowing NF-κB to sequester in the cytoplasm in an inactivated state [13–15]. As such it prevents subsequent nuclear translocation of phospho-NF-*κ*B to transactivate target genes. Therefore, the pre-application of BAY 11-7082 is expected to be highly effective in preventing acidic bile-induced NF-*κ*B activation and subsequent transcriptional activation of NF-*κ*B target genes. Interactions of NF-κB with oncogenic factors, such as *EGFR* and *STAT3* are particularly noteworthy because they have been previously cited to be active in HNSCC [16–18]. The upregulation of *WNT5A*, which is linked to the epithelial mesenchymal transition (EMT) process and cancer progression [19], has also been shown to be induced by NF-κB signaling [20], as is the rapid transcriptional activation of *TNF-α* [21].

Although post-application of BAY 11-7082, 15 min after acidic bile exposure, produces a minimal effect on acidic bile-induced *TNF-α* mRNA levels, it adequately suppresses the acidic bile-induced transcriptional activation of *RELA(p65), bcl-2, EGFR, STAT3, IL-6* and *WNT5A*. Immunofluorescence and western blot analysis shows cytoplasmic accumulation of p-NF-κB in post-treated MHPC in contrast to reduced nuclear p-NF-κB levels (Figures 1 and 2), implying that acidic bile constitutively activated NF-κB and that such activation persists after a short-term acidic bile exposure, allowing post-application of BAY 11-7082 to suppress its activity and subsequent oncogenic events. In contrast, the minimal effect of post-application on *TNF-α* suggests that its transcriptional activation occurs much more directly escaping late NF-κB inhibition.

The suppression of *bcl-2* and *IL-6* by either pre- and post-application of inhibitor is especially important because of *bcl-2* role in anti-apoptosis [22] and *IL-6* role as a cancer-related cytokine [23]. *bcl-2* is a transcriptional target of NF-κB, and its activity is significantly related to the anti-apoptotic role of NF-κB [24, 25]. In addition, *IL-6* previously found to be directly or indirectly induced by NF-κB, in HNSCC [26, 27]. The observation that the application of BAY 11-7082 either before or after acidic bile exposure is capable of inhibiting by a similar way the transcriptional levels of *bcl-2* and *IL-6*, support again the effectiveness of either early or late NF-κB inhibition in suppressing acidic bile-induced downstream anti-apoptotic and inflammatory pathways linked to carcinogenesis.

Our novel data from miRNA analysis show that either pre- or post-application of BAY 11-7082 can effectively prevent the acidic bile-induced deregulation of specific cancer-related miRNA markers, further supporting the observation that acidic bile-induced NF-κB activation may directly or indirectly regulate the expression of small regulatory molecules, miR-21, miR-155, miR-192, miR-375 and miR-451a [7, 9, 10].

Application of BAY 11-7082 either before or after acidic bile exposure induced a profound inhibition of miR-21 upregulation, was found to play an important role in head and neck cancer [28]. We are aware of previous observations demonstrating an independent association between NF-κB activation and up-regulation of oncogenic miR-21 [29, 30], and that *STAT3* may also be implicated in up-regulation of miR-21, in a manner of NF-κB-dependent *IL-6* up-regulation [31], supporting the view that inhibition of NF-κB, contributing to *STAT3* suppression, both lead to profound inhibition of miR-21.

Although pre-application of inhibitor suppresses the acidic bile-induced “downregulation” of miR-34a, the post-application does not, suggesting a mechanism of acidic bile-induced “downregulation” of this “tumor suppressor” miRNA marker that is complex [32, 33]. Similarly, although miR-375 is affected either by pre- or post-application of BAY 11-7082, post-application is found significantly less intense. Furthermore, pre-application of NF-κB inhibitor on miR-375 is also found clearly less intense compared to its effect on “oncomirs” miR-21 or miR-155. These observations again support a more complex mechanism of interaction in the acidic bile-induced deregulation of the “tumor suppressor” miR-375 [34–36]. In contrast, the observation that either pre- or post-application of NF-κB inhibitor results in profound inhibition of acidic bile-induced “downregulation” of “tumor suppressor” miR-451a, emphasizes that even late inhibition of NF-κB is capable of suppressing acidic bile-deregulation of a “tumor suppressor’ miRNA marker, we previously identified as related to laryngopharyngeal carcinogenesis [37].

Overall, the kinetic relationships between miRNA deregulation and NF-κB are highly complex. Although a full characterization of this network is beyond the scope of this report, interesting relationships are noted supporting future exploration.

Since systemic application of BAY 11-7082 causing certain toxicities, topical application of NF-κB inhibitors is a proposed approach. We have previously shown that topical co-administration of BAY 11-7082 on murine hypopharyngeal mucosa can prevent acidic bile induced molecular changes [6]. Based on the findings described in our current *in vitro* study, we are planning to pursue evaluation of pre and post topical application of BAY 11-7082 *in vivo,* using a similar protocol to the current *in vitro* study to provide temporal characteristics of NF-κB inhibition in blocking bile-induced oncogenic molecular events in our mouse model [10]. *In vitro* and *in vivo* applications of highly specific NF-κB inhibitors, such as BAY 11-7082 are necessary for the exploration of the central mechanistic role of NF-κB in acidic bile induced oncogenesis, suggesting that targeted intervention of this kind may have a preventive or therapeutic effect. Several pharmacologic and dietary inhibitors of NF-κB are considered promising therapeutic options with chemo-preventing or chemo-sensitizing properties in head and neck cancer [14, 26]. Curcumin, for example, is a natural turmeric supplement with known anti-oxidant, anti-inflammatory and anti-cancer properties, is considered pharmacologically safe [38]. Furthermore, it has previously shown to have potential chemo-preventive effects in head and neck malignancies [39], blocking NF-κB activation and halting the proliferation of cancer cells [40]. Thus, we have recently shown that *in vitro* co-administration of curcumin, a dietary NF-κB inhibitor is capable of preventing the acidic bile induced oncogenic changes, with results similar to BAY 11-7082 co-administration [12]. Determining the effectiveness of short term application of pharmacologic inhibition of NF-κB before and after acidic bile exposure, using highly specific inhibitors such as BAY 11-7082 will support future pre-clinical and clinical trials using other NF-κB inhibitors such as curcumin.

In conclusion, our novel findings show that short duration application of pharmacologic inhibitor of NF-κB 15 min before or after acidic bile exposure comparably prevents and suppresses its mRNA and miRNA oncogenic phenotypes in treated murine hypopharyngeal primary cells. In practical terms these observations strongly support the future clinical use of a topical NF-κB inhibitor in suppressing bile-induced oncogenic molecular events. Our data further provide a novel window of observation into the complex kinetics of an interesting mechanistic link between acidic bile and early neoplasia.

## MATERIALS AND METHODS

### Cell culture and treatment conditions

We cultured murine hypopharyngeal primary cells (MHPC) from Celprogen Inc. (Torrance, CA, USA), as previously described [6, 7]. Murine cells were selected because our prior *in vitro* explorations demonstrated that MHPC and human hypopharyngeal primary cells (HHPC) responded similarly to acidic bile with or without BAY 11-7082 [(E)-3-(4-methylphenylsulphonyl)-1-propenenitrile] [10]. Our proposed *in vitro* model would therefore facilitate future extension of our exploration to an already established *in vivo* murine model.

We performed the following repetitive procedures in parallel twice a day for 3 days: (i) acidic bile (pH 4.0) exposure; (ii) pre-application of BAY 11-7082 (pH 7.0); and (iii) post-application of BAY 11-7082 (pH 7.0) (Figure 6). Experiments were performed in triplicate.

(i) Acidic bile: The procedure included 7 minutes of exposure to a mixture of conjugated bile salts (400 μM) (Glycocholic acid:taurocholic acid:glycochenodeoxycholic acid:taurodeoxycholic acid:glycodeoxycholic acid:taurodeoxycholic acid at molar concentration 20:3:15:3:6:1) (Sigma, St. Louis, MO; Calbiochem, San Diego, CA, USA), in full growth medium (Dulbecco modified Eagle’s medium/F12 10% FBS, 1% pen/strep, Gibco^®^, NY, USA) brought to a pH 4.0 with 1M HCl (using a pH meter), as previously described [5, 8, 41, 42].

(ii) Pre-application of BAY 11-7082: The procedure included 15 minutes of applied BAY 11-7082 (10 μM) [43] (Calbiochem 2016 EMD Millipore Corporation; Germany) in full growth medium (Dulbecco modified Eagle’s medium/F12 10% FBS, 1% pen/strep, Gibco^®^, NY, USA) at pH 7.0. This NF-kB inhibitor was aspirated, replaced by serum free medium (KGM-2 SF, Gibco^®^, NY, USA) for 5, 10 or 15 minutes and followed by exposure to acidic bile. After acidic bile exposure (7 min) the media were removed and replaced by serum free media until the next exposure.

(iii) Post-application of BAY 11-7082: The procedure included first the acidic bile exposure. After acidic bile exposure (7 min) the media were aspirated and replaced by serum free medium (KGM-2 SF, Gibco^®^, NY, USA) for 5, 10 or 15 minutes. Then BAY 11-7082 (10 μM) was applied for 15 minutes in full growth medium (Dulbecco modified Eagle’s medium/F12 10% FBS, 1% pen/strep, Gibco^®^, NY, USA) at pH 7.0. After the application of BAY 11-7082 (7 min) the media were removed and replaced by serum free media until the next exposure.

Control groups for the NF-κB inhibitor vehicle (DMSO) included repetitive exposures for 10 minutes to full growth medium (Dulbecco modified Eagle’s medium/F12 10% FBS, 1% pen/strep, Gibco^®^, NY, USA) at pH 7.0. The media were removed and replaced by serum free media until the next exposure.

At the end of the treatment procedure, media were removed and cells or cell extracts were analyzed.

### Immunofluorescence assay

We performed an immunofluorescence assay to explore the effect of NF-κB inhibitor application 5, 10 and15 minutes before and after acidic bile-induced nuclear translocation of NF-κB transcription factor p65, phosphorylated at Ser536 [44], as we previously described [7].

Briefly, MHPCs were grown on slides (multiwall chamber slides; Lab-Tek^®^) and underwent treatment procedures, as described above. We used 1:65 of primary anti-NF-κB (rabbit polyclonal anti-phospho-p65 Ser536, AbD Serotec, BIO-RAD, CA, USA), and 1:500 dilutions of secondary anti-rabbit DyLight^®^488 (green; Vector Labs, USA). Prolong Gold Mountant with diamidino-phenylindole (ProLong^®^ Diamond Antifade Mountant with DAPI; Life Technologies, Thermo Scientific, MA, USA) was used for nuclear staining and mounting of cells (blue). The slides were examined using a Zeiss Confocal microscope and images were captured and analyzed using Zen imaging software from Carl Zeiss, microscopy (Germany). Total p-p65 (S536) expression levels in MHPC pre- and post-treated with NF-κB inhibitor and acidic bile alone were identified by fluorescence intensity (mean±SD bin count) from two independent images (≥10 cells) (Zen imaging software).

### Western Blotting

We performed Western blot analysis, as described previously [6, 7], to determine the nuclear and cytoplasmic protein expression levels of p-NF-κB (p65 S536) and bcl-2, respectively, on pre- and post-treated MHPC with NF-κB inhibitor BAY 11-7082 relative to acidic bile alone.

### Luciferase assay

We performed a luciferase assay in order to monitor the transcriptional activity of NF-κB in MHPC pre- and post-treated with pharmacologic inhibitor of NF-κB, BAY 11-7082 relative to acidic bile alone. We used Firefly Luciferase Assay system (Promega Corporation, Madison, WI, USA), Lipofectamine^®^ 2000 (Invitrogen™), and pGL4.32[luc2P/NF-κB-RE/Hygro] Vector, encoded with the firefly luciferase reporter gene (luc2P) driven by five copies of an NF-κB enhancer element, and control vector (pGL4.27[luc2P/minP/Hygro]), and in accordance with the manufacturer’s procedure. Equal number of cells were transfected with NF-κB or control luciferase vector. The treatment was performed 24 hours after transfection. We performed triplicate assays for each treatment condition. The cells were treated once with acidic bile alone (7 min), pre-application with BAY 11-7082 (15 min), post-application with BAY 11-7082 (15 min), and corresponding controls, as described above in “cell culture and treatment conditions”. Luminescence was measured using a luminometer (Infinite^®^ M1000 PRO, TECAN) and i-control™ software. We expressed NF-κB activity as ratios of mean values [values for NF-κB reporter (NF-κB-luc2P), against the mean value for control (luc2P)] calculated in treated MHPC for each condition.

### Quantitative real time PCR

We isolated total RNA (RNeasy mini kit; Qiagen Inc., CA, USA) from MHPC exposed to acidic bile alone, pre-treated and post-treated with BAY 11-7082 groups, and controls, to evaluate the transcriptional levels of *RELA (p65), bcl-2, TNF-α, EGFR, STAT3, WNT5A* and *IL-6*, using quantitative real time polymerase chain reaction (qPCR) analysis and specific primers for mouse genome, as previously described [8, 10]. Glyceraldehyde 3-phosphate dehydrogenase (GAPDH) was used as a reference housekeeping gene (QuantiTect Primers Assays; Qiagen) [10]. We performed assays in 96-well plates, in triplicate for each sample, and data were analyzed by CFX96™ software. Relative mRNA expression levels were estimated for each target gene relative to reference gene (ΔΔ*C*t). (Data were obtained from three independent experiments)**.**

### miRNA analysis

We performed miRNA analysis to determine the expression levels of “oncomirs” and ‘tumor suppressor” miRNA specific markers in MHPC exposed to acidic bile alone, pre-treated or post-treated with BAY 11-7082, and controls. Specifically, we analyzed the expression of “oncomirs” miR-21, miR-155, and miR-192, and “tumor suppressors” miR-34a, miR-375, and miR-451a, previously linked to laryngopharyngeal cancer [35–37, 45–47], using specific primers for target-miRNAs of mouse genome (miScript Primer Assays, Qiagen^®^, KY, USA) and normalization control small RNA [snRNA RNU6B (RNU6-2), as previously described [7, 10]. We estimated relative expression levels (target miRNA/RNU6B) for each specific miRNA marker, in each experimental and control group (CFX96™ software; Bio-Rad, CA, USA) (Data were obtained from three independent experiments).

### Statistical analysis

Statistical analysis was performed using GraphPad Prism 7.0 software and ONE-WAY ANOVA (Friedman or Kruskal-Wallis; Dunn’s multiple analysis test; *p* values < 0.05) as well as *t*-test analysis (multiple comparisons by Holm-Sidak) in order to reveal any evidence of statistically significant reductions of protein, mRNA or miRNA expression levels in groups pre- or post-treated with NF-κB inhibitor, compared to acidic bile alone and control treated groups. We performed Pearson correlation to estimate the correlation coefficient between expression levels of the analyzed genes and miRNA markers in the studied groups (*p* values<0.05).
